# Increased Length of Awareness of Assisted Reproductive Technologies Fosters Positive Attitudes and Acceptance among Women

**DOI:** 10.22074/ijfs.2015.4603

**Published:** 2015-12-23

**Authors:** Chelsea Fortin, Susanne Abele

**Affiliations:** 1 Department of Psychology, Miami University, Oxford, OH, USA; 2 Department of Obstetrics and Gynecology, Cleveland Clinic, Cleveland, OH, USA

**Keywords:** Assisted Reproductive Technology (ART), Attitudes, Awareness, Infertility, *In Vitro* Fertilization (IVF)

## Abstract

**Background:**

The field of infertility medicine has witnessed a surge of scientific developments in recent years, but research on public attitudes towards infertility treatments has remained minimal. This study examined the social and demographic factors that affect women’s attitudes towards assisted reproductive technology (ART) in general, as well as their opinions of specific issues related to ART.

**Materials and Methods:**

This cross-sectional study was conducted from March 2011 to April 2011 by means of an online survey administered to a sample of 287 women.

**Results:**

Women with a longer length of awareness of ART had significantly greater attitudinal favorability towards ART. Political affiliation was also significantly related to general attitudes, as well as several specific aspects of ART issues.

**Conclusion:**

The results of this study suggest that several factors influence attitudes that women hold in regards to ART. Identifying some of these factors serves as a crucial starting point for devising strategies to increase public acceptance of ART.

## Introduction

Over the past half-century, societal changes in the United States have markedly altered typical childbearing patterns. For a number of reasons, including the penalties associated with taking time off work and the enormous expense of raising a child, an increasing number of women have chosen to wait to have children. Each year, women are having their first child later than ever before. Whereas birth rates for U.S. women who are in their twenties have steadily declined each year, the rate has soared for those in their thirties and forties (1). As more and more women choose to delay motherhood, the frequency of women experiencing infertility issues has continued to rise. Today, a shocking one in six U.S. couples is affected by infertility. In 1982, 6.6 million women in the U.S. received infertility treatments and in 2002, this number increased to 7.3 million (2). Globally, it has been estimated that as many as 48.5 million couples worldwide are infertile (3).

Since the first child conceived through *in vitro* fertilization (IVF) was born in 1978 (4), there has been an explosion of advancements in the area of assisted reproductive technology (ART). ART is the collective term used to refer to the medical procedures involving the laboratory manipulation of eggs and sperm that increase the chances that a woman will achieve pregnancy. The most commonly performed ART procedure is IVF, but the term also includes preimplantation genetic diagnosis (PGD) and the use of donor eggs (5).

The recent advancements in ART have expanded the choices available to both physicians and patients, and have simultaneously created new ethical issues. Some of these issues concern the medical risks associated with infertility techniques, such as the high incidence of multiple pregnancies-which increases the risk of cesarean section, preterm labor and delivery, low birth weight and death (6). Others involve redefining what constitutes a family and whether there are negative implications for children brought into a family by means of certain ART techniques. Recently, many of these issues have revolved around questions of patient access to infertility treatments.

Empirical research on these newly conceived ethical issues has not kept pace with this rapidly evolving field of medicine. Published series have mainly focused on the obstetric outcome and development of children conceived via infertility treatments. There is limited research on the public’s perceptions of these treatments, yet it is essential that health care providers are aware of these perceptions, so that they can practice ART in a fashion that is acceptable to the public.

ART has always been controversial on religious grounds, and thus, religion is likely a factor that influences women’s attitudes towards this subject. Research suggests that highly religious individuals tend to hold more traditional views on marriage and family patterns (7). Those with strong religious beliefs also tend to hold more conservative views towards genetic testing (8) and have ethical concerns with ART procedures (9, 10). Surprisingly, however, the Catholic Church remains the only major world religion that explicitly forbids the use of IVF (11).

Past research suggests that political affiliation influences attitudes towards ART and other reproductive health issues. In fact, a recent study found that political affiliation was one of the strongest predictors of approval of IVF use for nontraditional women (e.g. single women, homosexuals), with approval being higher amongst Democrats than Republicans (12). Shreffler et al. (9) demonstrated that those with liberal social-political views are less likely than their conservative counterparts to have ethical concerns with ART. Similar findings have been documented in the realm of embryonic stem cell research (13), abortion (14) reproductive genetic testing (15) and posthumous reproduction (16). These findings parallel the core values that divide party lines: Republicans tend to place more value on the traditional family structure, whereas Democrats are often more liberal in regards to reproductive health issues (17-20).

To our knowledge, no previous research has explored a possible link between attitudes and women’s length of awareness of infertility treatments. It is reasonable to expect a relationship to exist between these variables based on a concept known as the mere exposure effect, a phenomenon in which the mere repetition of an individual’s exposure to a stimulus enhances his/her attitude towards the stimulus (21). This idea that familiarity leads to liking has been demonstrated across a variety of stimulus domains- including attitudes towards mental illness (22), organ transplantation (23), assisted living (24), newborn screening programs (25), biotech foods (26) and epilepsy (27)-but heretofore not in the realm of infertility medicine.

The goal of this study was to investigate the social and demographic factors related to women’s attitudes towards ART, including political affiliation, education, ethnicity, religion, income, and age. Other factors more specific to the study of infertility treatments, such as length of awareness of ART, having participated in ART techniques, knowing someone else who has undergone ART, exposure to ART through the media, and current health status (fertile or infertile), were also examined. Finally, women’s knowledge of infertility treatments was examined to identify any potential misconceptions of ART.

## Materials and Methods

The Institutional Review Board for the Use of Human Subjects in Research at Miami University approved the research plan and the survey content on March 17, 2011. Participants were all recruited through Qualtrics, an online professional survey firm (www.qualtrics.com). The recruitment pool is managed by Qualtrics’ panel partner, Clearvoice Research, which comprises a census-representative panel of over one million members around the world. We recruited only panelists from the United States for this study. Past medical research has demonstrated the effective use of this company to recruit participants (28-30).

The company pulls a sample in quota groups and then uses simple randomization to produce a representative sample. The average panelist response rate (determined by clicks per invitation sent) is 20%. Many procedures are in place to confirm the identity of respondents, including verification of United States Postal Services (USPS) postal addresses, using flash cookies, and tracking internet protocol (IP) addresses. The research company maintains full records on panelist activity and limits panelists to one completed survey every ten days. Survey respondents are rewarded with a cash value amount, ranging from $1.00 to $20.00, based on the length of the survey and the target audience. This reward is then credited to the respondent’s account. Once the respondent’s account value exceeds $10.00, he/she can redeem for his/her selection of gift certificates or prepaid debit cards.

This cross-sectional study excluded men in an attempt to thoroughly examine many factors that impact attitudes towards ART rather than looking at any gender differences that might exist. Of the 341 women that were invited to take the survey and subsequently clicked the invitation, 324 agreed to the consent form and completed the survey (response rate: 95%). Data from 37 respondents were eliminated because they either did not fit into the specified age group and gender, they did not complete the entire survey, or they completed the survey too quickly for their results to be considered reliable (i.e. under five minutes). Thus the final sample size was 287. Table 1 summarizes the demographic and clinical characteristics of respondents.

All of the women surveyed fell into one of three age groups: 24 to 29 (n=84), 34 to 39 (n=106), and 44 to 49 (n=97). These age groups were chosen, so that distinctive differences between age groups could be identified. Aside from these age and gender restrictions, no other qualifiers were used, and qualifying participants were selected at random. All participants provided consent before being able to access the survey, and were debriefed upon completion of the survey.

After obtaining approval from the Institutional Review Board at Miami University in Oxford, OH, the survey was developed and pilot-tested. The survey instrument was formulated on the basis of a review of the literature related to attitudes towards ART. The survey was pilot-tested on ten undergraduate students in March 2011 at Miami University. The subjects were asked to complete the survey and provide feedback on the questions. They were also asked to record the amount of time required to complete the survey. The feedback obtained was used to develop the final version of the survey; however, no results were obtained and/or used.

The finalized survey was then made available on the Qualtrics online survey system from March to April 2011. It consisted of three main sections: questions pertaining to attitudes towards ART, demographics, and knowledge of ART. All questions, with the exception of one question ("Where did you hear about these treatments?"), were mutually exclusive; in other words, participants were only allowed to select one answer of those available. The survey took participants approximately 15 minutes to complete.

A series of 36 attitudinal questions were used to measure respondent opinions on the ethical aspects of ART. Participants were instructed to gauge their opinion on a nine-point Likert-type scale, with responses ranging from "strongly disagree" (1) to "strongly agree" (9). A subset of six of the attitudinal questions assessed respondents’ general attitudes towards infertility treatments. Six additional subscales were constructed to examine attitudes towards specific details of ART. For each subscale, items were selected for inclusion based on content analysis and subsequent factor reliability analysis. Table 2 depicts the composition of each subscale, as well as the Cronbach’s alpha (α), which is a measure of inter-item reliability.

**Table 1:** Demographics and clinical characteristics of participants


Demographic	n	%

Age (Y)		
24-29	84	29
34-39	106	37
44-49	97	34
Educational attainment		
High school or lower	56	20
Some college	84	29
Associates degree	39	14
Bachelor’s degree	73	25
Master’s degree	25	9
Doctoral degree	10	4
Religion		
Muslim	4	1
Christian (non-Catholic)	143	50
Roman Catholic	76	27
Jewish	13	5
Hindu	4	1
Buddhist	7	2
None	29	10
Other	11	4
Frequency of church attendanceNever	115	40
Religious holidays only	78	27
Monthly	35	12
Weekly	51	18
DailyAnnual household income	8	3
Less than $25,000	55	19
$25,000-$50,000	85	30
$50,000-$100,000	101	35
Above $100,000	31	11
Prefer not to answer	15	5
Ethnicity		
Caucasian	236	82
African American	15	5
Hispanic	12	4
Asian	18	6
Other	6	2
Political affiliation		
Democrat	102	36
Republican	67	23
Independent	63	22
Other	42	2
Prefer not to answer	13	15
Length of awareness of ART		
None	3	1
Less than one year	8	3
One year	19	7
Five years	85	30
Ten years or longer	172	60
Current health status		
Fertile	136	47
Infertile	62	22
Unknown	89	31
Recipient of ART		
Yes	12	4
No	271	94
Know recipient of ART		
Yes	116	60
No	171	40
Heard of Octomom?		
Yes	187	35
No	100	65
Heard of Frieda Birnbaum?(60 year old recipient of IVF)		
Yes	109	62
No	178	38


ART; Assisted reproductive technology and IVF; *In vitro* fertilization.

**Table 2:** Attitudinal subscales


Subscale	Items	Cronbach’s alpha (α)

**General**	I am in favor of infertility treatments in general	0.859
	Infertility treatments are tampering with nature*	
	Infertility treatments are tampering with nature…; and therefore, make me uneasy so I would not consider them for myself*	
	Infertility treatments are tampering with nature…; and therefore, are unethical and should not be performed*	
	The benefits of infertility treatments outweigh the risks	
	Infertility treatments carry unknown consequences*	
**Sperm donation**	If a man’s sperm are not viable, it is acceptable for him to use sperm donation	0.804
	It is acceptable for a young, healthy man to donate his sperm	
	Sperm donor bank are acceptable for homosexuals who want to have a child	
	Sperm donor banks are acceptable for parents to choose a father who is particularly intelligent	
**Egg donation**	If a woman’s eggs are not viable, it is acceptable for her to use egg donation	0.844
	It is acceptable for a young, healthy woman to donate her eggs	
**IVF**	IVF is an acceptable treatment for couples with infertility problems	0.703
	Preimplantation genetic diagnosis is a procedure of genetic testing performed on an embryo prior to implantation. I believe that this is an acceptable procedure in order to select a healthy, compatible embryo that can cure a sibling suffering from some disease	
	IVF is an acceptable option for couples with serious genetic diseases to select embryos that do not carry the defective gene	
	For fertile couples, it is acceptable to use IVF to choose the sex of their child	
**Selective embryo reduction**	Selective embryo reduction is a procedure in which the number of fetuses is reduced in a pregnancy involving more than one fetus. I believe that this practice is appropriate	0.613
	Selective embryo reduction is appropriate if the baby and/or mother are threatened	
**Regulation of ART**	I trust those in charge of new developments to act in society’s interests in regards to infertility treatments	0.573
	I trust the regulatory system for infertility treatments to keep pace with scientific advancements	
	Regulations on infertility treatments are too relaxed*	
	The rules governing infertility treatments are well enforced	
**Accessibility of ART**	There should be an age limit for infertility treatments*	0.771
	Single women should have access to infertility treatments	
	Individuals with criminal charges or a history of sexual offense should have access to infertility treatments	
	Individuals with diseases/disabilities that may interfere with their ability to parent a child should have access to infertility treatments	
	Sperm donor banks are acceptable for homosexuals who want to have a child	
	IVF is an acceptable option for couples with serious genetic diseases to select embryos that do not carry the defective gene	
	For fertile couples, it is acceptable to use IVF to choose the sex of their child	
	Sperm donor banks are acceptable for parents to choose a father who is particularly intelligent	


ART; Assisted reproductive technology, IVF; *In vitro* fertilization and *; Reverse scored.

Respondent knowledge of infertility treatments was measured with 22 multiple-choice items. Each question had one correct response. These questions tested knowledge of a variety of aspects of ART, such as procedural information, the incidence of infertility, and the financial burden of treatment (see supplemental section at www. Ijfs.ir). The remaining questions pertained to demographics and other clinical factors specific to ART (Table 1).

Following data collection, a series of statistical analyses were run using the Statistical Package for the Social Sciences (SPSS) program, version 18 (SPSS Inc., Chicago IL). An analysis of variance (ANOVA) was run to examine associations between each demographic variable and attitudes on the ethics of ART, as well as knowledge of ART. The paired samples t test was used to identify relationships between women’s attitudes of sperm and egg donation. A series of analysis of covariance (ANCOVA) was run to determine if any other factors covaried with respondent ethnicity and also to determine whether or not the two factors, political affiliation and length of awareness, covaried with each other. Finally, we studied the relationship between length of awareness and general attitudes by means of a general linear regression.

## Results

**Fig.1:**
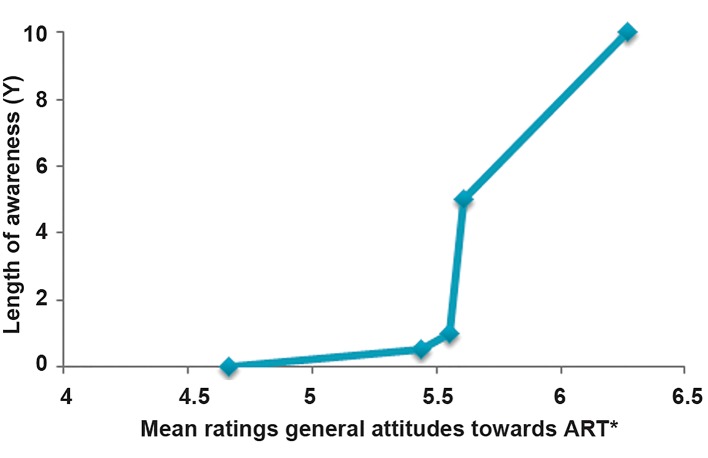
General attitude toward ART depending on length of awareness. *; Measured on a 9-point Likert-type scale (1=lowest; 9=highest) and ART; Assisted reproductive technology.

The measure of general attitudes towards ART was significantly related to two factors. First, a simple linear regression using length of awareness as a predictor and general attitudes as the dependent variable revealed a significant regression weight (b=0.188) for the length of awareness [t (285)=3.23, P<0.001]. Hence, participants’ general attitudes towards ART became progressively more positive as their length of awareness of ART increased (Fig .1). As depicted in table 3, political affiliation was also significantly related to general attitudes towards ART [F (2, 229)=7.24, P=0.001]. In general, all affiliations were relatively supportive of ART (M>5 in all three groups); however, Democratic women were the most supportive and Republican women were the least supportive.

Political affiliation and length of awareness were unrelated [F<1, n.s.]. An ANCOVA, using political affiliation as a factor and length of awareness as a covariate, yielded a significant result of the covariate, length of awareness [F (1,228)=8.08, P<0.005], and a significant result of political affiliation [F (2,228)=8.15, P<0.000]. Therefore, while both length of awareness and political affiliation affect women’s general attitude towards ART, both of these effects are parallel, and the effect of political affiliation cannot be explained by length of awareness.

A series of ANOVAs were run to examine the relationship between each individual attitudinal subscale and each of the demographic/clinical factors listed in table 1. Many of the factors varied significantly within specific attitudinal subscales. Table 3 shows the mean attitudinal ratings corresponding to the statistically significant ANOVAs. Significant F values indicate that the average attitudinal rating for a specific subscale differed significantly across the individual categories of that particular demographic/clinical factor. The remaining ANOVAs not shown in table 3 were not significant. We wished to further analyze women’s attitudes towards specific aspects of the regulation of ART, which is depicted in table 4.

The attitudinal questions regarding gamete donation revealed significant differences in women’s opinions of sperm and egg donation. Sperm donors were rated as significantly more likely to donate only for the money [t(286)=-8.38, P=0.000], but egg donors were rated as significantly more likely to later regret their decision to donate for both psychological and medical reasons [t(286)=6.88, P=0.000 and t(286)=7.06, P=0.000, respectively]. Also, for both egg and sperm donation, women were significantly more supportive of an individual donating their gametes than they were of an individual utilizing donated material [t(286)=-2.06, P=0.040 and t(286)=-3.33, P=0.001, respectively] (Table 5).

Over four-fifths (83%) of respondents who had heard of IVF (n=263) underestimated the incidence of twins with IVF. When asked what fraction of infertility patients eventually has a baby after treatment, only 25% of participants correctly selected 70% of patients. Only 17% of women knew that the level of malformations is higher in ART children. Similarly, over half (54%) of participants overestimated the percentage of infertility cases due to female factors as compared to the percentage due to male factors. Among those who had heard of IVF, 95% overestimated the frequency of IVF as the treatment of choice for infertility. And finally, respondents who underestimated the price of a single cycle of IVF (M=5.93, SD=2.42) were significantly less likely to agree that the cost of infertility treatments is unreasonable than those who did not underestimate this value (M=6.80, SD=1.98) [ F(1,261)=4.71, P=0.031].

**Table 3:** ANOVA results of mean (SD) attitudinal ratings within factors influencing attitudes towards ART


	Political affiliation^a^			F	P
Attitudes toward	Republican	Democrat	Independent		

General attitudes	5.4 (1.9)	6.4 (1.6)	6.1 (1.6)	7.24	0.001
Sperm donation	5.3 (2.2)	6.5 (1.7)	6.0 (2.0)	7.20	0.000
Egg donation	6.3 (2.3)	7.1 (2.0)	7.1 (2.0)	3.32	0.038
IVF	4.8 (1.8)	5.8 (1.6)	5.4 (1.8)	7.41	0.001
Regulation of ART	4.4 (1.7)	5.4 (1.4)	5.0 (1.6)	8.04	0.000
Accessibility of ART	3.9 (1.7)	5.0 (1.4)	4.6 (1.6)	9.58	0.000
	**Frequency of church attendance^b^**			
	**Frequently**	**Infrequently**	**Never**		
Sperm donation	5.0 (2.3)	6.0 (1.9)	6.3 (1.9)	8.79	0.000
Egg donation	4.8 (2.6)	5.4 (1.9)	5.5 (1.9)	7.33	0.001
IVF	4.8 (2.0)	5.4 (1.5)	5.5 (1.7)	3.93	0.021
Embryo reduction	4.9 (2.4)	5.8 (2.0)	5.9 (1.8)	5.29	0.006
Accessibility of ART	3.9 (1.9)	4.5 (1.5)	4.8 (1.4)	7.10	0.000
	**Religion^c^**			
	**Catholic**	**Non-Catholic Christian**			
Embryo reduction	6.0(1.6)	5.1(1.5)		4.28	0.040
	**Know participant of ART?**			
	**yes**	**No**			
Regulation of ART	4.6 (1.6)	5.1 (1.5)		7.01	0.009
	**Participant of ART?^d^**			
	**yes**	**No**			
Regulation of ART	3.8 (4.9)	2.0 (1.6)		13.66	0.000
	**Ethnicity^e, f^**			
	**Caucasian**	**Non-Caucasian**			
Regulation of ART	4.9(1.6)	5.3(1.6)	-	4.26	0.040
Accessibility of ART	4.4(1.6)	4.9(1.5)	-	4.73	0.030


^a^; The categories "Other" and "Prefer not to answer" and "None" were excluded from analysis, ^b^; For analysis purposes, women who reported attending monthly or only on religious holidays were considered to have "infrequent" attendance. Those who said that they attend either daily or weekly were classified as having "frequent" attendance, ^c^; Religion was dichotomized into Catholic vs. non-Catholic Christian due to insufficient participants in other religious groups, ^d^; The number of women who reported being a participant in ART (n=12) is too low to make any solid conclusions, ^e^; Women were divided into two ethnic groups-Caucasian and non-Caucasian-due to insufficient participants in other ethnic groups, ^f^; A series of ANCOVAs was run to determine if any other factors covaried with ethnicity [between-subjects factor: ethnicity (Caucasian, non-Caucasian); covariates: education, religion, political affiliation, frequency of church attendance, and length of awareness of ART] revealed that none of these measures could explain the effect of ethnicity (all Ps>.20). ART; Assisted reproductive technology and IVF; *In vitro* fertilization.

**Table 4:** Attitudes towards regulation of ART


**Attitudes toward**	Mean	SD

I trust those in charge of new developments to act in society’s interests in regards to infertility treatments	4.97	2.21
I trust the regulatory system for infertility treatments to keep pace withscientific advancements	5.15	2.13
Regulations on infertility treatments are too relaxed*	4.95	2.04
The rules governing infertility treatments are well enforced	4.69	1.81


*; Reverse scored and ART; Assisted reproductive technology.

**Table 5:** Attitudes towards gamete donation


Attitudes toward	n	Mean	SD	t	P

If a woman’s eggs are not viable, it is acceptable for her to use egg donation	287	6.71	2.31	1.39	0.167
If a man’s sperm are not viable, it is acceptable for him to use sperm donation	297	6.60	2.34		
It is acceptable for a young, healthy woman to donate her eggs	287	6.91	2.21	-0.15	0.878
It is acceptable for a young, healthy man to donate his sperm	287	6.92	2.26		
Most egg donors only donate their eggs for the money	287	5.46	2.27	-8.38	0.000
Most sperm donors only donate their sperm for the money	287	6.29	2.25		
It is likely that an egg donor would later regret her decision to donate her eggs for psychological reasons	287	4.46	2.20	6.88	0.000
It is likely that a sperm donor would later regret his decision to donate his sperm for psychological reasons	287	3.63	2.15		
It is likely that an egg donor would later regret her decision to donate her eggs for medical reasons	287	4.07	2.20	7.06	0.000
It is likely that a sperm donor would regret his decision to donate his sperm for medical reasons	287	3.32	2.10		


## Discussion

The overall general attitude towards ART was significantly related to two demographic factors: length of awareness of ART and political affiliation. This study is the first to identify a significant tie between the length of time that individuals are aware of ART and their attitudes towards these treatments. It is possible that this observed outcome is a manifestation of the mere exposure effect; that merely being exposed to infertility treatments is enough to increase acceptance and augment positive attitudes towards these treatments (21).

But why does this connection between length of awareness of ART and favorability of ART exist in the first place? Several researchers have attempted to explain the reasoning behind the link between familiarity and acceptance. Diamantopoulos et al. (27) studied attitudes towards epileptic individuals and concluded that people tend to be fearful of the things that they do not know or understand, so being familiar with a disorder naturally increases the degree of tolerance towards it. Perhaps those who have not been exposed to the topic of infertility treatments feel uneasy because they lack a basic understanding of these treatments, which hinders any opportunity for acceptance.

A similar theory proposes that familiarity influences stigma (25). Based on this model, those who have been aware of ART for a longer length of time might be less likely to endorse stigmatizing attitudes towards ART. This highlights the importance of informing the public of these treatments, so that stigma can be reduced and public acceptance facilitated.

The finding that political affiliation was significantly related to the general measure of attitudes towards ART as well as five of the specific attitudinal subscales indicate that this demographic factor is a major predictor of attitudes towards ART. In each of these instances, Republican women were less favorable towards ART than both Democratic and Independent women-results that further validate previous findings (8, 9, 14). This finding might reflect the effect of Republican women holding more conservative views generally, rather than being specifically induced by categorizing oneself as Republican. Regardless, it is important to discover what changes can be made to the presentation of ART, so that it is accepted by all political parties.

The observed attitudinal differences between Catholics and non-Catholic Christians were not congruent with what might be expected based on traditional religious orthodoxy. It was surprising to find that Catholic women were actually significantly more supportive towards many of the specific aspects of ART than non-Catholic Christian women. As has been previously documented, however, inconsistencies do exist between official religious discourse and the individual beliefs of followers (31, 32). Thus, perhaps the more appropriate indicator of one’s religiosity is frequency of church attendance. When viewed in this manner, it becomes apparent that there is a significant inverse relationship between religiosity and several of the specific measures of attitudes towards ARTnamely, attitudes towards sperm donation, egg donation, IVF, selective embryo reduction, and accessibility of ART.

The observation that there were significant differences in participant attitudes towards egg and sperm donation indicates that women do not view these two procedures as equal. The apparent gender discrepancy may indicate that social stigmas affect women’s opinions on these issues. Women also hold differing opinions towards gamete donation depending on whether an individual is donating or receiving a gamete. Again, it is possible that using donated gametes-but not being a donor of gametes-is a procedure that is stigmatized. Both of these findings are research questions that should be explored further.

Age was not significantly related to any of the attitudinal subscales. This finding was not unexpected, as previous studies of this association have been varied and inconsistent. A large survey on the public’s perceptions of infertility treatments conducted in six European countries, the USA, and Australia reported that opinions varied little among age groups (33). Similarly, Sigillo et al. (12) found no association between age and attitudes towards IVF for nontraditional women. On the other hand, Shreffler et al. (9) found that women under age 30 and women beyond 40 had higher ethical concerns than women in their thirties.

The lack of sufficient participants in certain categories (e.g. religious affiliation, ethnicity and being a participant of ART) is a limitation in our study.

A large portion of our respondents were Caucasian and well-educated. A larger, more diverse pool of participants would likely yield data that permit a clearer evaluation of national opinion. Our study is also limited due to the fact that the survey was administered online, which renders it prone to the limitations associated with internet research, including technical difficulties and uncertain representativeness of selected samples.

Because of the socioeconomic and ethical issues raised by ART, an awareness of the various public attitudes surrounding ART has important implications for many specific sectors. Medical professionals must be especially cognizant of these attitudes in light of the public’s concern for the ethics of medicine. A comprehensive understanding of public perceptions of ART is essential for all medical professionals, but particularly for those who practice reproductive techniques.

Our research highlights a need to inform the public in the realm of infertility medicine, so that any misconceptions can be eliminated or prevented-an undertaking that can only be realized with the support of the medical field. The high likelihood of overestimating and underestimating on many of the knowledge items indicates that the public is rather misinformed on some aspects of ART. The finding that those who underestimated the cost of IVF were less likely to agree that the cost of IVF is unreasonable illustrates the direct influence that misconceptions can have on one’s attitudes. This research should help healthcare professionals to educate the public about ART, so that misunderstandings do not hinder public acceptance of these treatments.

This line of research also has practical implications for legislators, and should help direct them towards making informed decisions about future ART policies. Our results revealed that women are not extremely trusting of the regulatory system, and tend to disagree that the rules governing infertility treatments are well enforced. Furthermore, it appears that those who are more familiar with, and have had more exposure to, ART are actually less supportive of the regulatory system (e.g. those who have undergone infertility treatments, those who know a participant of infertility treatments, and those who are infertile). It is essential that authorities continually gauge public opinion to uphold the public’s endorsement of a field that is constantly evolving.

## Conclusion

Our study identified key factors that influence the attitudes that women hold in regards to ART. For the first time, a link has been established between the length of time that a woman has been aware of ART, and her general attitudes towards ART. Age did not appear to be a significant factor; however, political affiliation and religion were significantly associated with women’s attitudes towards ART. Identifying some of the factors associated with decreased approval of infertility treatments serves as a crucial starting point for formulating strategies for wider public understanding.

## Supplementary PDF


