# Supplementary material for: Increased Length of Awareness of Assisted Reproductive Technologies Fosters Positive Attitudes and Acceptance among Women
Source: Int J Fertil Steril. 2015 Dec 23;9(4):452–64. doi: 10.22074/ijfs.2015.4603 (PMC4841309; doi:10.22074/ijfs.2015.4603)
Supplement: Supplementary file 1 [file Int-J-Fertil-Steril-9-452-s01.pdf]

Int J Fertil Steril
INTERNATIONAL JOURNAL OF FERTILITY & STERILITY
ISSN: 2008-076x eISSN: 2008-0778

www.ijfs.ir

Supplementary Information for

Increased Length of Awareness of Assisted Reproductive Technologies Fosters Positive Attitudes and Acceptance among Women

Chelsea Fortin, M.D.[1, 2]*, Susanne Abele, Ph.D.[1]

1. Department of Psychology, Miami University, Oxford, OH, USA
2. Department of Obstetrics and Gynecology, Cleveland Clinic, Cleveland, OH, USA

*Corresponding Address: Department of Obstetrics and Gynecology, Cleveland Clinic, 9500 Euclid Ave, Desk A81, Cleveland, OH, USA 44195-0001
Email: fortinc@ccf.org

Attitudes towards Assisted Reproductive Technologies Questionnaire

With the following items we would like to assess what people think about infertility treatments. There is no correct answer; select one response for each of the following items, indicating how much you agree with each of the statements, with (1) meaning you strongly disagree and (9) meaning you strongly agree.

Strongly disagree   Strongly agree
1 2 3 4 5 6 7 8 9

1. I am in favor of infertility treatments in general

1 2 3 4 5 6 7 8 9

2. *In vitro* fertilization (IVF) is as an acceptable treatment for couples with fertility problems

1 2 3 4 5 6 7 8 9

3. Infertility treatments are tampering with nature

1 2 3 4 5 6 7 8 9

…, and therefore; make me uneasy so I wouldn't consider them for myself

1 2 3 4 5 6 7 8 9

…, and therefore; are unethical and should not be performed

1 2 3 4 5 6 7 8 9

4. Preimplantation genetic diagnosis is a procedure of genetic testing performed on an embryo prior to implantation. I believe that this is an acceptable procedure in order to select a healthy, compatible embryo that can cure a sibling suffering from some genetic disease

1 2 3 4 5 6 7 8 9

5. If a woman's eggs are not viable, it is acceptable for her to use egg donation

1 2 3 4 5 6 7 8 9

6. It is acceptable for a young, healthy woman to donate her eggs

1 2 3 4 5 6 7 8 9

7. Most egg donors only donate their eggs for the money

1 2 3 4 5 6 7 8 9

8. It is likely that an egg donor would later regret her decision to donate her eggs for psychological reasons

1 2 3 4 5 6 7 8 9

9. It is likely that an egg donor would later regret her decision to donate her eggs for medical reasons

1 2 3 4 5 6 7 8 9

10. If a man's sperm are not viable, it is acceptable for him to use sperm donation

1 2 3 4 5 6 7 8 9

11. It is acceptable for a young, healthy man to donate his sperm

1 2 3 4 5 6 7 8 9

12. Most sperm donors only donate their sperm for the money

1 2 3 4 5 6 7 8 9

13. It is likely that a sperm donor would later regret his decision to donate his sperm for psychological reasons

1 2 3 4 5 6 7 8 9

14. It is likely that a sperm donor would later regret his decision to donate his sperm for medical reasons

1 2 3 4 5 6 7 8 9

15. Selective embryo reduction is a procedure in which the number of fetuses is reduced in a pregnancy involving more than one fetus. I believe that this practice is appropriate

1 2 3 4 5 6 7 8 9

16. Selective embryo reduction is appropriate if the baby and/or mother are threatened

1 2 3 4 5 6 7 8 9

17. The cost of infertility treatments is unreasonable/burdensome

1 2 3 4 5 6 7 8 9

18. Infertility is a disability/medical condition

1 2 3 4 5 6 7 8 9

19. Insurance should not cover infertility treatment because it is an elective procedure (a planned, non-emergency procedure)

1 2 3 4 5 6 7 8 9

20. There should be an age limit for infertility treatments

1 2 3 4 5 6 7 8 9

21. Single women should have access to infertility treatments

1 2 3 4 5 6 7 8 9

22. Individuals with criminal charges or a history of sexual offense should have access to infertility treatments

1 2 3 4 5 6 7 8 9

23. Individuals with diseases/disabilities that may interfere with their ability to parent a child should have access to infertility treatments

1 2 3 4 5 6 7 8 9

24. IVF is an acceptable option for couples with serious genetic diseases to select embryos that do not carry the defective gene

1 2 3 4 5 6 7 8 9

25. For fertile couples, it is acceptable to use IVF to choose the sex of their child

1 2 3 4 5 6 7 8 9

26. Sperm donor banks are acceptable for homosexuals who want to have a child

1 2 3 4 5 6 7 8 9

27. Sperm donor banks are acceptable for parents to choose a father who is particularly intelligent

1 2 3 4 5 6 7 8 9

28. The benefits of infertility treatments outweigh the risks

1 2 3 4 5 6 7 8 9

29. Infertility treatments carry unknown consequences

1 2 3 4 5 6 7 8 9

30. I trust those in charge of new developments to act in society's interests in regards to infertility treatments

1 2 3 4 5 6 7 8 9

31. Infertility treatments represent a major advance in science

1 2 3 4 5 6 7 8 9

32. Regulations on infertility treatments are too relaxed

1 2 3 4 5 6 7 8 9

33. I trust the regulatory system for infertility treatments to keep pace with scientific advancements

1 2 3 4 5 6 7 8 9

34. The rules governing infertility treatments are well enforced

1 2 3 4 5 6 7 8 9

With the following items we would like to assess how much people know infertility treatments. Please answer each question to the best of your ability. If you are unsure of an answer, make your best educated guess.

Infertility is recognized as a disease by leading medical societies. It is defined by the World Health Organization (WHO) as “the inability of a couple to achieve conception or to bring a pregnancy to term after a period of regular, unprotected, intercourse.” How long do you think that period is (33)[1]?

- ○ 6 months to a year
- ○ 1 to 2 years
- ○ Over 2 years
- ○ Unable to conceive at all

Compared with children who are conceived naturally, the intelligence quotient (IQ) of children conceived through infertility treatments is (34):

- ○ Much higher
- ○ Higher
- ○ Roughly the same (Correct answer)
- ○ Lower
- ○ Much lower

Compared with children who are conceived naturally, the height and weight of children conceived through infertility treatments is (34):

- ○ Much higher
- ○ Higher
- ○ Roughly the same (Correct answer)
- ○ Lower
- ○ Much lower

Compared with children who are conceived naturally, the level of malformations among children conceived through infertility treatments is (34):

- ○ Much higher
- ○ Higher (Correct answer)
- ○ Roughly the same
- ○ Lower
- ○ Much lower

Compared with children who are conceived naturally, the verbal performance of children conceived through infertility treatments is (34):

- ○ Much higher
- ○ Higher
- ○ Roughly the same (Correct answer)
- ○ Lower
- ○ Much lower

Compared with children who are conceived naturally, the motor development of children conceived through infertility treatments is (34):

- ○ Much higher
- ○ Higher

1. This question was later omitted from analyses due to the ambiguity of the answer choices

○ Roughly the same (Correct answer)
○ Lower
○ Much lower

Compared with children who are conceived naturally, the behavior and temperament of children conceived through infertility treatments is (34):

○ Much higher
○ Higher
○ Roughly the same (Correct answer)
○ Lower
○ Much lower

Compared with parents of children who are conceived naturally, the stress level of parents of children conceived through infertility treatments is (34):

○ Much higher
○ Higher
○ Roughly the same (Correct answer)
○ Lower
○ Much lower

How many couples experience infertility (2)?

○ 1 in 100
○ 1 in 50
○ 1 in 10
○ 1 in 6 (Correct answer)
○ 1 in 2

In what percentage of states in the US is there some type of insurance coverage for infertility treatments (35)?

○ 0%
○ 30% (Correct answer)
○ 75%
○ 100%

The percentage of infertility cases due to female factors is _____ compared to the percentage of infertility cases due to male factors (36)

○ Much higher
○ Higher
○ Roughly the same (Correct answer)
○ Lower
○ Much lower

What fraction of infertility patients eventually has a baby after treatment (37)?

○ 10%
○ 30%
○ 50%

○ 70% (Correct answer)
I have heard of the Nadya Suleman (Octomom) case

○ Yes
○ No

I have heard of Frieda Birnbaum, the 60-year-old woman who used IVF

○ Yes
○ No

I have heard of reproductive tourism in which women travel abroad for fertility treatments

○ Yes
○ No

Have you ever heard of IVF?

○ Yes
○ No

What occurs during the process of IVF (28)?

○ A single sperm is injected into a single egg
○ A woman is given drugs to induce ovulation
○ One or more eggs are combined with a sample of sperm in a Petri dish (Correct answer)
○ A sample of sperm is placed into the female reproductive tract

IVF accounts for ___% of all infertility treatment in the United States (38)

○ 5 (Correct answer)
○ 10
○ 25
○ 50
○ 75

One out of every ____ babies born in the United States is conceived using IVF (39)

○ 10
○ 100 (Correct answer)
○ 1000
○ 10,000
Approximately what percentage of IVF pregnancies results in twins (40)?

○ Less than 20%
○ 20%
○ 30%
○ 40%
○ 50% (Correct answer)

Approximately what percentage of IVF pregnancies results in triplets or more (41)?

- ○ 2%
- ○ 5% (Correct answer)
- ○ 10%
- ○ 20%

If a woman under 40 years old undergoes an IVF treatment cycle today where up to three fertilized eggs are placed in her womb, what chance do you think she has of having a baby as a result, compared with a fertile couple trying to have a baby through regular unprotected sexual intercourse during one month? Would you think the IVF patients' chances were…(33)?

- ○ Much higher
- ○ Higher
- ○ Roughly the same (Correct answer)
- ○ Lower
- ○ Much lower

Approximately, what is the average price of one cycle of IVF in the United States (42)?

- ○ $500
- ○ $1000
- ○ $10,000 (Correct answer)
- ○ $20,000

An ectopic pregnancy is one in which the fertilized egg implants outside the uterus and must be aborted. Compared to women who conceive naturally, the risk of ectopic pregnancy in mothers using IVF is (43)

- ○ Much higher
- ○ Higher (Correct answer)
- ○ Roughly the same
- ○ Lower
- ○ Much lower

When will a woman's physician recommend in IVF (44)?

- ○ If she has been diagnosed with unexplained infertility
- ○ If her Fallopian tubes have been blocked
- ○ If other techniques such as fertility drugs or artificial insemination have not been successful
- ○ All of the above (Correct answer)
- ○ None of the above
